# Quantitative imaging of tongue kinematics during infant feeding and adult swallowing reveals highly conserved patterns

**DOI:** 10.14814/phy2.14685

**Published:** 2021-02-06

**Authors:** Catherine W. Genna, Yiela Saperstein, Scott A. Siegel, Andrew F. Laine, David Elad

**Affiliations:** ^1^ International Board Certified Lactation Consultant Woodhaven NY USA; ^2^ Department of Biomedical Engineering Columbia University New York NY USA; ^3^ School of Medicine/School of Dental Medicine Stony Brook University Suffolk County NY USA; ^4^ Department of Biomedical Engineering Tel‐Aviv University Tel‐Aviv Israel

**Keywords:** Bolus swallowing, Bottle‐feeding, Breastfeeding, Submental Ultrasound, Tongue motility

## Abstract

Tongue motility is an essential physiological component of human feeding from infancy through adulthood. At present, it is a challenge to distinguish among the many pathologies of swallowing due to the absence of quantitative tools. We objectively quantified tongue kinematics from ultrasound imaging during infant and adult feeding. The functional advantage of this method is presented in several subjects with swallowing difficulties. We demonstrated for the first time the differences in tongue kinematics during breast‐ and bottle‐feeding, showing the arrhythmic sucking pattern during bottle‐feeding as compared with breastfeeding in the same infant with torticollis. The method clearly displayed the improvement of tongue motility after frenotomy in infants with either tongue‐tie or restrictive labial frenulum. The analysis also revealed the absence of posterior tongue peristalsis required for safe swallowing in an infant with dysphagia. We also analyzed for the first time the tongue kinematics in an adult during water bolus swallowing demonstrating tongue peristaltic‐like movements in both anterior and posterior segments. First, the anterior segment undulates to close off the oral cavity and the posterior segment held the bolus, and then, the posterior tongue propelled the bolus to the pharynx. The present methodology of quantitative imaging revealed highly conserved patterns of tongue kinematics that can differentiate between swallowing pathologies and evaluate treatment interventions. The method is novel and objective and has the potential to advance knowledge about the normal swallowing and management of feeding disorders.

## Graphical abstract

Tongue kinematics was objectively quantified from ultrasound imaging during feeding. Tongue and palate contours were traced in each frame of the video; tongue outlines were registered with respect to the hard anterior palate; and tongue motility characteristics were determined from intersections with polar coordinates in subsequent frames. Differences in tongue kinematics during breast‐ and bottle‐feeding are demonstrate in the same infant. The method detected improvement of tongue motility after frenotomy interventions and revealed absence of posterior tongue peristalsis in infant with dysphagia. It demonstrated tongue peristaltic‐like movements in both anterior and posterior segments in an adult during water bolus swallowing.

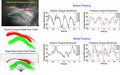

## 1 INTRODUCTION

The tongue muscle is an active organ in the oral cavity with crucial roles in feeding, speech, and breathing (Matsuo & Palmer, 2008; Stone et al., 2018; Xing et al., 2019). It is an intrinsic skeletal muscle with the origin and insertion at the same point in the root. Embryonic development of the tongue starts at weeks 4–5 from the mesoderm and maturation begins at about 6 months when infants start chewing and swallowing solid food, and continues during 12 to 36 months (Iskander & Sanders, 2003; Miller et al., 2003; Delaney & Arvedson, 2008; Rosero Salazar et a., 2020). The tongue's 3D motion and deformation are controlled by the intrinsic (genioglossus, transverse and vertical, superior and inferior longitudinal) and extrinsic tongue muscles (hyoglossus, styloglossus, palatoglossus) and the tethering to the mandible via the lingual frenulum (Mills et al., 2019; Thibodeau & Patton, 2007). The dynamic performance of the tongue in healthy and diseased humans is complex, but here we focus on the functional role of the tongue in human feeding.

Infant feeding on the breast is important for normal growth and lifelong wellness, and accordingly, breastfeeding is recommended for at least the first year of life (Binns et al., 2016; Eidelman, 2012; Genna, 2017). In preparation for breastfeeding the infant needs to latch‐on to the breast and draw the nipple‐areola complex into its mouth with the nipple tip extended near the hard‐soft palate junction (HSPJ) (Jacobs et al., 2007; Neville, 2001; Woolridge, 1986). Successful breastfeeding requires dynamic synchronization between the oscillation of the infant's mandible, rhythmic motility of the tongue, and the breast's milk ejection reflex that drives maternal milk toward the nipple outlets. During suckling, the infant compresses the areola region and the underlying tissue with the tongue interposed between the lower gum and breast. Sub‐atmospheric oral pressures are generated via the oscillating mandible and pulsating tongue (Geddes et al., 2008; Kent et al., 2008; Woolridge, 1986). The infant efficiently coordinates suckling, swallowing, and breathing via the central nervous system without apnea or hypoxia (Bu'Lock et al., 1990; Goldfield et al., 2006; Koenig et al., 1990). The tongue muscle plays a key functional role in the regulation of optimized extraction and swallowing of human milk from the breast.

Many infants are also fed with man‐made bottles and nipples, whether with human milk extracted by hand or a breast pump, or with modified animal milks or plant protein solutions. Though the dynamic performance of the infant during bottle‐feeding seems to be similar to that of breastfeeding (Smith et al., 1985) there are significant differences (Hernandez & Bianchini, 2019). Artificial nipples are more rigid than human nipples and do not reshape themselves to fit the infants’ mouth in response to the feeding action (Goldfield et al., 2006). Moreover, the spontaneous undulating motion of the infant's tongue observed during suckling on the breast is impeded during bottle‐feeding (Bu'Lock et al., 1990). While milk flow during breastfeeding depends on milk production in the breast and infant demand imposed by suckling, the man‐made bottle‐nipple system allows for continuous milk flow with minimal tongue and mandibular motions (Matsubara & Inoue, 2019). Increased and uncontrolled milk delivery during bottle‐feeding results in more frequent breathing interruptions (Taki et al., 2010) which leads to episodes of oxygen desaturation (Baeza et al., 2017; Chen et al., 2000; Hammerman & Kaplan, 1995).

Tongue‐tie or ankyloglossia is an anomaly where the frenulum is attached too far forward along the tongue or is too thick or too stiff, and as a result, tongue mobility may be restricted or impaired. Tongue‐tie is a major cause for breastfeeding difficulties with a prevalence of 4–12% of USA newborns (O'Shea et al., 2017; Walsh et a., 2017). Lingual frenotomy is a minor surgical procedure in which an incision in the frenulum releases the excess tethering of the tongue. Numerous prospective, retrospective, and randomized controlled studies, all based on subjective observations, have reported improvement in breastfeeding outcomes after lingual frenotomy (Bellinger et al., 2018; Berry et al., 2012; Buryk et al., 2011; Dollberg et al., 2014; Emond et al., 2014; Geddes, Langton, et al., 2008; Ghaheri et al., 2017; Ramoser et al., 2019). The complexity of tongue development and its functional role during breastfeeding led to controversies regarding diagnostic criteria, treatment indications, interventions (e.g., frenotomy), as well as the monitoring and evaluation of clinical interventions. While opinions and definitions have shifted from morphology toward more functional aspects, the absence of objective tools to measure functional parameters for grading the level of physiological restriction has led to growing debates and the potential for overdiagnosis and unnecessary surgeries (O'Shea et al., 2017; Walsh et al., 2017; Walsh & Tunkel, 2017). An objective analysis of tongue motility can assist in identifying infants with truly restricted tongue movements and reveal confounding conditions.

Swallowing is a multidimensional complex process of transporting food from the oral cavity to the stomach while the airways are protected. It is divided into oral, pharyngeal, and esophageal stages and involves the tongue, mandible, hyoid, pharynx, larynx, and esophagus (Matsuo & Palmer, 2008; Sasegbon & Hamdy, 2017). The tongue is the main active organ in the oral stage of swallowing which is described differently for liquid and solid food. During the intake of liquid, the cupped tongue gathers a bolus in the oral cavity (i.e., preparatory phase), then quickly propels it into the oropharynx (i.e., propulsive phase). While eating solid food, the tongue transports the food to the molars for processing by the teeth and saliva, and when suitable for swallowing, it is moved to the midline of the tongue and propelled into the oropharynx. Dysphagia is the medical term for swallowing disorders that may involve the oral cavity, pharynx, esophagus or the gastroesophageal junction. Oropharyngeal dysphagia is defined as difficulty or inability to transport a bolus safely and effectively from the oral cavity to the esophagus (Cabib et al., 2016; Ortega et al., 2017; Sasegbon & Hamdy, 2017).

The tongue muscle is the main active organ in the oral stage of swallowing; however, objective detection of its dynamics is a difficult task and many methods were utilized over the years for the measurement of tongue motion during speech or swallowing (Hiiemae & Palmer, 2003). Cine‐Radiology was utilized in the early 1950 s to study swallowing mechanisms in the mouth and pharynx (Ardran & Kemp, 1951). It ceased in humans in the late 1980 s due to concerns about radiation exposure. Instead, videofluorography became the gold standard for diagnosis of the mouth, pharynx, and esophagus during swallowing (Hiiemae & Palmer, 1999; Martin‐Harris & Jones, 2008; Matsuo & Palmer, 2016). Ultrasound imaging of the tongue during swallowing and speech begun in the early 1980 s and has been widely used since then (Huckabee et al., 2015; Shawker et al., 1983; Stone, 2005; Watkin, 1999). In the late 1990 s, MRI was also explored as an acquisition modality to study tongue motility (Stone et al., 2001). Non‐imaging methods include electropalatography which uses multiple sensors to measure the contact force between the tongue and hard palate, and the electromagnetic articulometer, which uses tiny transmitter coils attached to the tongue surface to measure the movement of specific locations on the tongue surface (Hiiemae & Palmer, 2003). It should be noted that the measurement of tongue motion during food swallowing is more complicated than during linguistic protocols.

The literature is rich with verbal descriptions of the role of tongue kinematics during breastfeeding (Bu'Lock et al., 1990; Geddes, Kent, et al., 2008; Goldfield et al., 2006; Kent et al., 2008; Koenig et al., 1990; Neville, 2001; Woolridge, 1986) and swallowing a bolus of liquid (Casas et al., 2002; Neufeld & Lieshout, 2014; Steele & Van Lieshout, 2009). However, these studies were based on subjective observations of ultrasound video clips or manual measurements of small numbers of subjects. More continuous tracking of the instantaneous tongue upper outline was possible by implementing the active contour model (e.g., snakes) on ultrasound movies recorded during swallowing (Akgul et al., 1999; Chi‐Fishman, 2005; Iskarous, 2005; Li et al., 2005a, 2005b; Parthasarathy et al., 2005; Stone, 2005). In addition, polar coordinates were imposed for local analysis of tongue dimensions (Bressmann et al., 2005). In recent studies of tongue movement during speech, ultrasound recordings with a head‐transducer support system were analyzed to provide local tongue movement and velocity along the polar coordinates (Berti et al., 2016; de Boer & Bressmann, 2016; Bressmann et al., 2016, 2017; Rastadmehr et al., 2008; Tang et al., 2012). Similar procedures were also used to explore tongue displacement and intra‐oral transit time during liquid swallowing (Berti et al., 2016; Soares et al., 2015). Nevertheless, knowledge on tongue kinematics during feeding is still incomplete, and quantitative objective methodologies are still unavailable in the clinic.

Recently, we utilized similar methods to extract the tongue and palate contours from ultrasound video clips and developed an objective method to quantify the infant's tongue kinematics during breastfeeding (Elad et al., 2014). More recently, we implemented methods previously used to analyze the periodicity of murine uterine horn contractions to enable the analysis of the instantaneous spectrum of motility (Zhang et al., 2019). Here, we employed the objective analysis to explore the instantaneous kinematics at any location along the tongue during several conditions of infant feeding and adult swallowing of a liquid bolus.

## 2 METHODS

**Figure 1:**
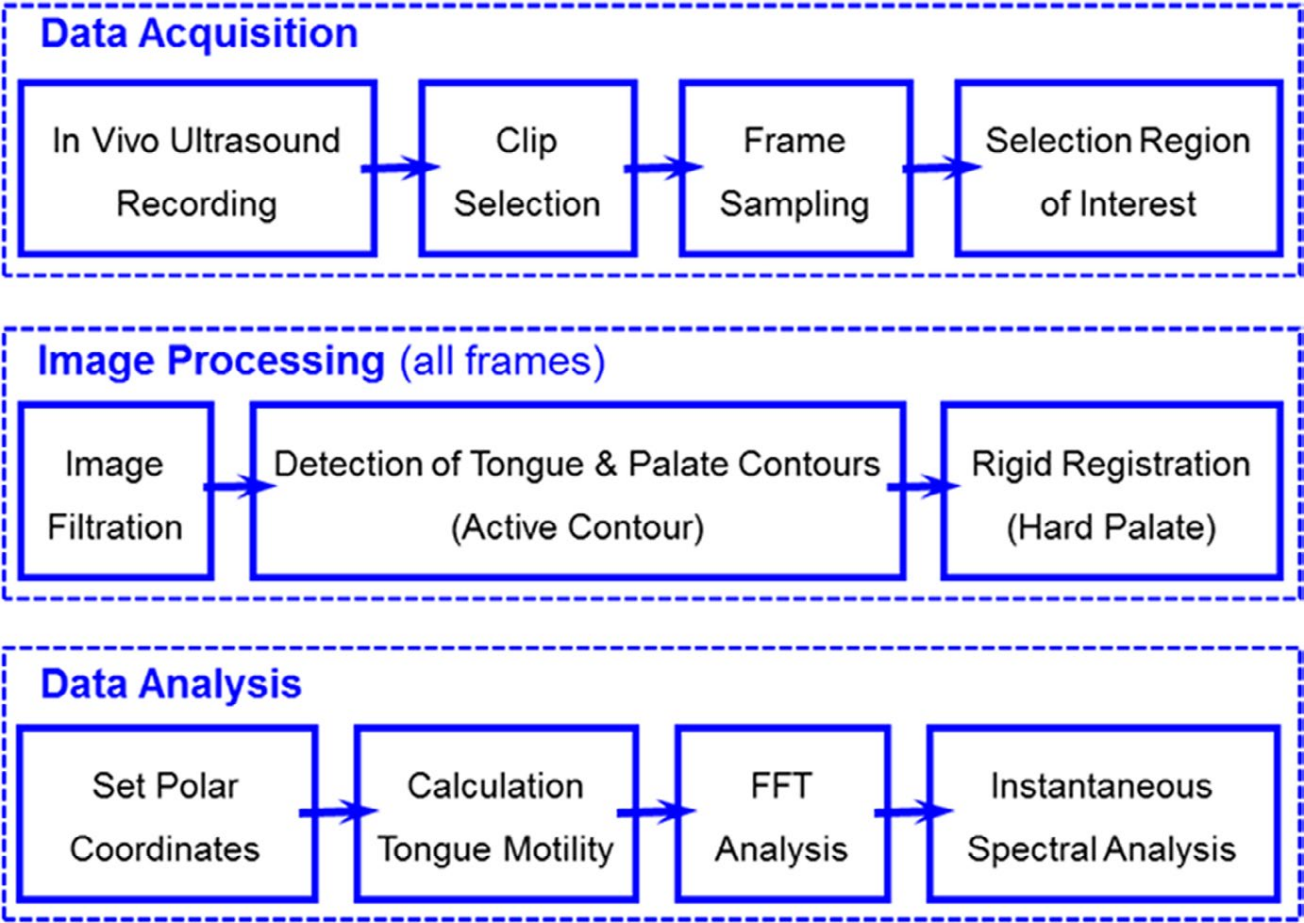
Flow chart for analysis of tongue kinematics pattern from video clips of submental ultrasound imaging during breastfeeding

The tongue kinematics was analyzed from in vivo submental ultrasound video clips. The computational methods were similar to those used in our previous studies where we converted video clips of medical images into time‐dependent biological data that can be analyzed in the time‐frequency‐space domains for the enhancement of physiological knowledge (Elad et al., 2014; Eytan et al., 1999; Gora et al., 2016, 2018; Meirzon et al., 2011; Zhang et al., 2019). The experimental and objective computational approach to explore tongue kinematics during infant feeding or adult swallowing is schematically shown in the flow chart depicted in Figure 1. It was composed of the following stages: (a) In vivo data acquisition and image preparation; (b) Image processing and tracking of the tongue and palate contours; and, (c) Analysis of tongue spatial motility and time–frequency spectral analysis of tongue kinematics with respect to the hard palate.

### 2.1 Subjects and Experimental protocol

We employed the methodology of analyzing tongue kinematics in to a variety of feeding conditions: one infant with torticollis during breast‐ and bottle‐feeding, one infant with tongue‐tie breastfeeding pre‐ and post‐frenotomy, one infant with restrictive superior labial frenum (lip‐tie) breastfeeding pre‐ and post‐frenotomy, one infant with dysphagia breastfeeding, and one adult swallowing a bolus of water. The mid‐sagittal section of the oral cavity was acquired submentally with the General Electric NextGen LOGIQ e R7 compact system using the E8C‐RS endocavitary transducer. It is a relatively small microconvex array transducer with a long handle and multi‐frequency capability (4–10 MHz) for far‐field imaging. The study was approved by the Columbia University IRB committee (#AAAR‐5986 and #AAAR‐7823). All participants signed informed consent, parents signed on behalf of their infant. The infant's cooperation was considered assent.

Data acquisition during infant breastfeeding was conducted, while the infant was held by the mother in a comfortable “cradle hold” nursing position. The transducer was placed under the infant's chin (i.e., the submental approach) with minimal interference to the infant's attachment to the breast. Several 3‐second ultrasound cine‐clips were recorded once the infant began nutritive sucking. Data acquisition during adult swallowing was conducted while sitting upright in a comfortable position. Recording of ultrasound images started a few seconds before the swallowing of a bolus of water and finished about 1 second after its completion. The recorded data were saved as AVI movie files.

### 2.2 Analysis of Tongue Kinematics

**Figure 2:**
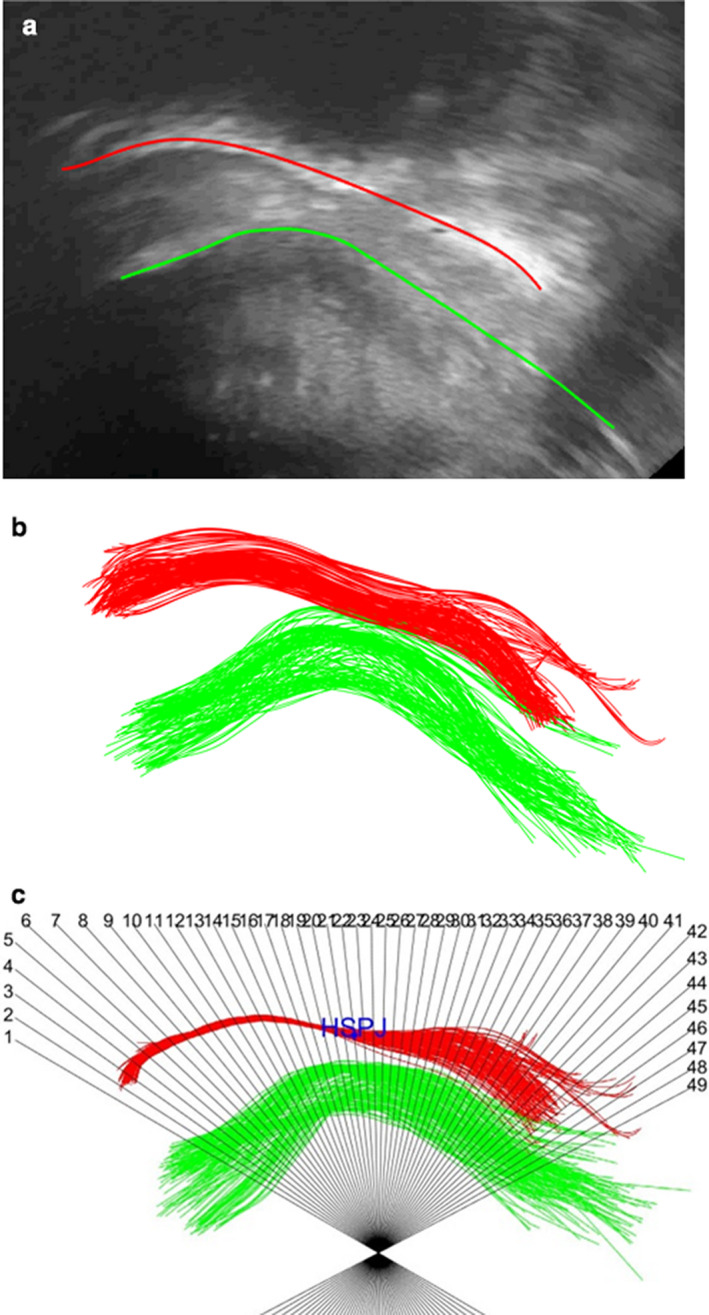
Analysis of tongue motility: (a) Tongue and palate tracking on a single image, (b) Contours of tongue and platae from all video frames before registration, (c) Contours of tongue and platae from all video frames after registration with polar coordinates

First, we selected a section of the recorded ultrasound video with visible parts of the upper tongue and the palate. For analysis of breastfeeding we selected about five cycles, while for swallowing we selected a single cycle. The frame rate, which is needed for the dynamic analysis, was determined using the FrameRate function. Then, the sequence of frames was sampled into BMP images and the region that contains the upper tongue and palate was selected for further processing. Noise reduction and image improvement were performed with an anisotropic diffusion filter. In the next step, the contours of the tongue and palate were traced on all the images by employing the vector field convolution method (i.e., snakes or active contour model) (Elad et al., 2014; Gora et al., 2018; Zhang et al., 2019) that generated accurate smooth contours within a few iterations (Figure 2a,b). This procedure requires manual initialization by marking a few points on the first image. In order to remove noise due to breathing and movements of the mother, infant, and technician, we registered all images with respect to the anterior part of the hard palate, which does not deform during breastfeeding (Elad et al., 2014).

In order to determine the tongue motility with respect to the anterior hard palate, we imposed a system of polar coordinates with the origin under the tongue outline (Figure 2c). The local tongue motility was determined from the intersections of the instantaneous tongue contour in subsequent frames. The vectors obtained from tongue intersection with each of the polar coordinates provided the time‐dependent motility of the tongue about this coordinate. The set of vectors of intersections with all the polar coordinates provided the special motility of the tongue with respect to the hard palate which does not deform during breastfeeding.

The frequency spectrum of the tongue motility was explored by the application of a fast Fourier transform (FFT) to the curves of tongue motility at any given location. This analysis also provided the dominant frequency of the tongue. Since the motility signal is not necessarily a steady signal, we also determined the periodicity characteristic by applying an auto‐covariance analysis. We used the MATLAB function “xcov” that returns the auto‐covariance sequence of an array, which in the present study stands for the periodicity of the signal. This analysis is displayed in this work at given axial locations along the tongue.

Since the data of tongue motility vary with frequency and location along the tongue, we also employed a wavelet analysis to amplify the signals internal content. For this purpose, we utilized the Magnitude‐Squared Wavelet Coherence method, which is based on the power spectral densities of the input signals. We used the MATLAB function "wcoherence" with a Morlet type window / basis. Since this method compares the coherence between a pair of signals, we computed the scaled coherence between pairs of motility curves about the polar lines in the anterior (e.g., lines 5 & 7), middle (e.g., lines 8 & 12), and posterior tongue (e.g., lines 17 & 21).

## 3 RESULTS

**Figure 3:**
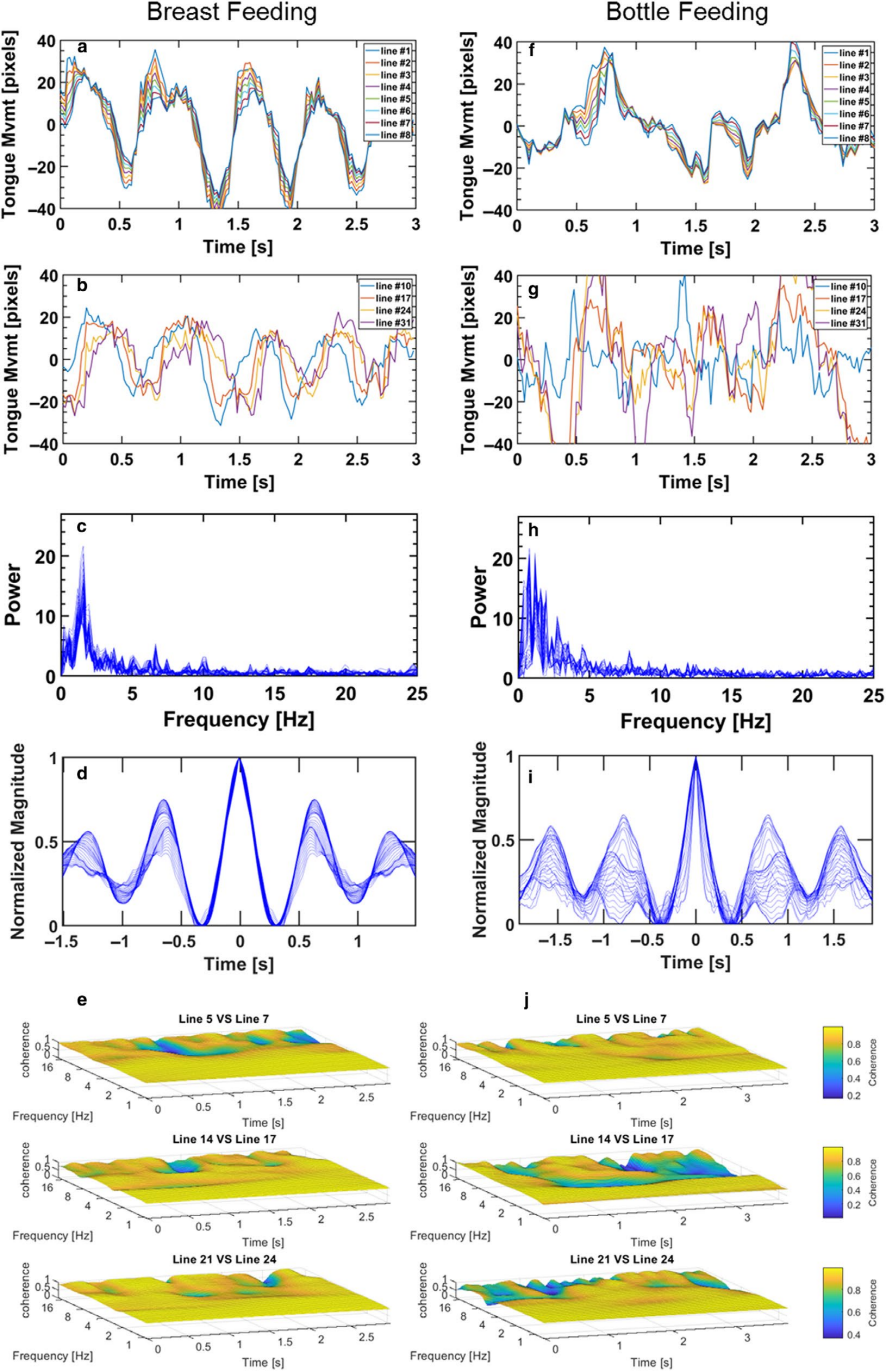
Tongue motility of infant with torticollis during breast feeding (a through e) and bottle‐feeding (f through i). (a,f) Motility of the anterior tongue during breast‐ and bottle‐feeding, (b,g) Motility of the posterior tongue during breast‐ and bottle‐feeding, (c,h) Frequency distribution of tongue motility during breast‐ and bottle‐feeding, (d,i) Periodicity (autocovariance) of tongue motility during breast‐ and bottle‐feeding (e,j) Coherence of pairs of motility signals during breast‐ and bottle‐feeding at in the anterior (lines 5 & 7), middle (lines 8 & 12) and posterior tongue (lines 17 & 21)

We applied the objective analysis to tongue kinematics from ultrasound movies recorded during infant feeding and adult swallowing. The tongue motility results for a 4.6‐weeks‐old infant with left torticollis and airway instability while breastfeeding are demonstrated in Figure 3 (a through e). The movement of the anterior and posterior sections of the tongue are depicted in Figure 3a,b for specific polar lines. The tongue motility pattern of this infant is similar to that of a healthy infant, but less rhythmic (Elad et al., 2014). The anterior section demonstrates a rigid motion, while in the posterior part the lines are shifted with time which is typical for a peristaltic pattern. The frequency spectrum about all the polar coordinates after the registration of all the images is depicted in Figure 3c. The dominant frequency for the whole tongue is 1.56 Hz. The pattern of local periodicity for each polar line is presented in Figure 3d). Wavelet analysis with a Morlet type window (Figure 3e) demonstrates the time‐frequency pattern by the scaled coherence between pairs of motility curves about the polar lines for the anterior (lines 5 & 7), middle (lines 14 & 17), and posterior tongue (lines 21 & 24).

The same infant was also studied during bottle‐feeding with expressed human milk and the resultant analysis is shown in Figure 3f‐j. The anterior part of the tongue is still moving like a rigid body but with a fluctuating periodicity (Figure 3f), while the peristaltic motion of the posterior part is almost absent (Figure 3g). The frequency spectrum does not show a dominant frequency (Figure 3h) as shown for breastfeeding. The irregular periodicity is depicted in Figure 3i and clearly demonstrates the significant difference in tongue motility between breast‐ and bottle‐feeding. The Morlet wavelet analysis shows significant variability of high frequencies in the range of 4–8 Hz during bottle‐feeding (Figure 3j). The ultrasound movies with the tracked outline of the tongue are provided in the supplemental Video S1.

**Figure 4:**
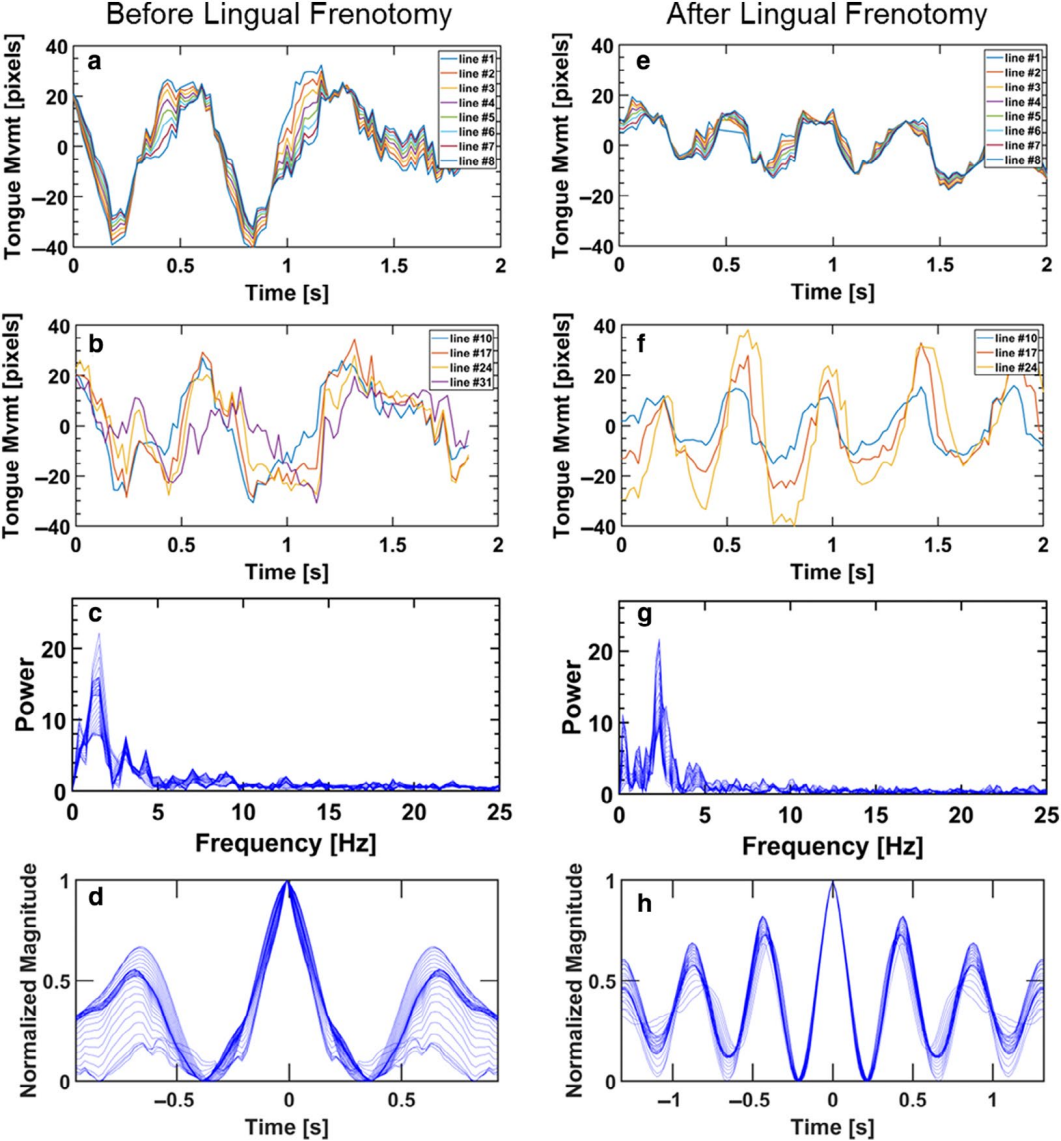
Tongue motility of tongue‐tied infant before.(a through d) and after lingual frenotomy (e through h). (a,e) Motility of the anterior tongue pre‐ and post‐frenotomy, (b,f) Motility of the posterior tongue pre‐ and post‐frenotomy, (c,g) Frequency distribution of tongue motility pre‐ and post‐frenotomy, (d,h) Periodicity (autocovariance) of tongue motility pre‐ and post‐frenotomy

The next examples were conducted to explore the outcome of surgical interventions in cases of tongue‐tied and lip‐tied infants. First, we analyzed the tongue motility of a 3‐week‐old tongue‐tied infant with a restrictive lingual frenulum, while breastfeeding before and after the frenotomy intervention (Figure 4). The movies with the tracked outlines of the tongue and palate are included in the supplemental Video S2. The tongue motility before the surgical intervention is chaotic with a smeared frequency spectrum and unstable periodicity (Figure 4a‐d). The pattern of tongue motility immediately post‐frenotomy is depicted in Figure 4e‐h, which conveys significant improvement to become similar to that of a healthy infant. The periodicity of the anterior and posterior tongue segments is smooth and repeatable while the frequency spectrum reveals a dominant frequency.

**Figure 5:**
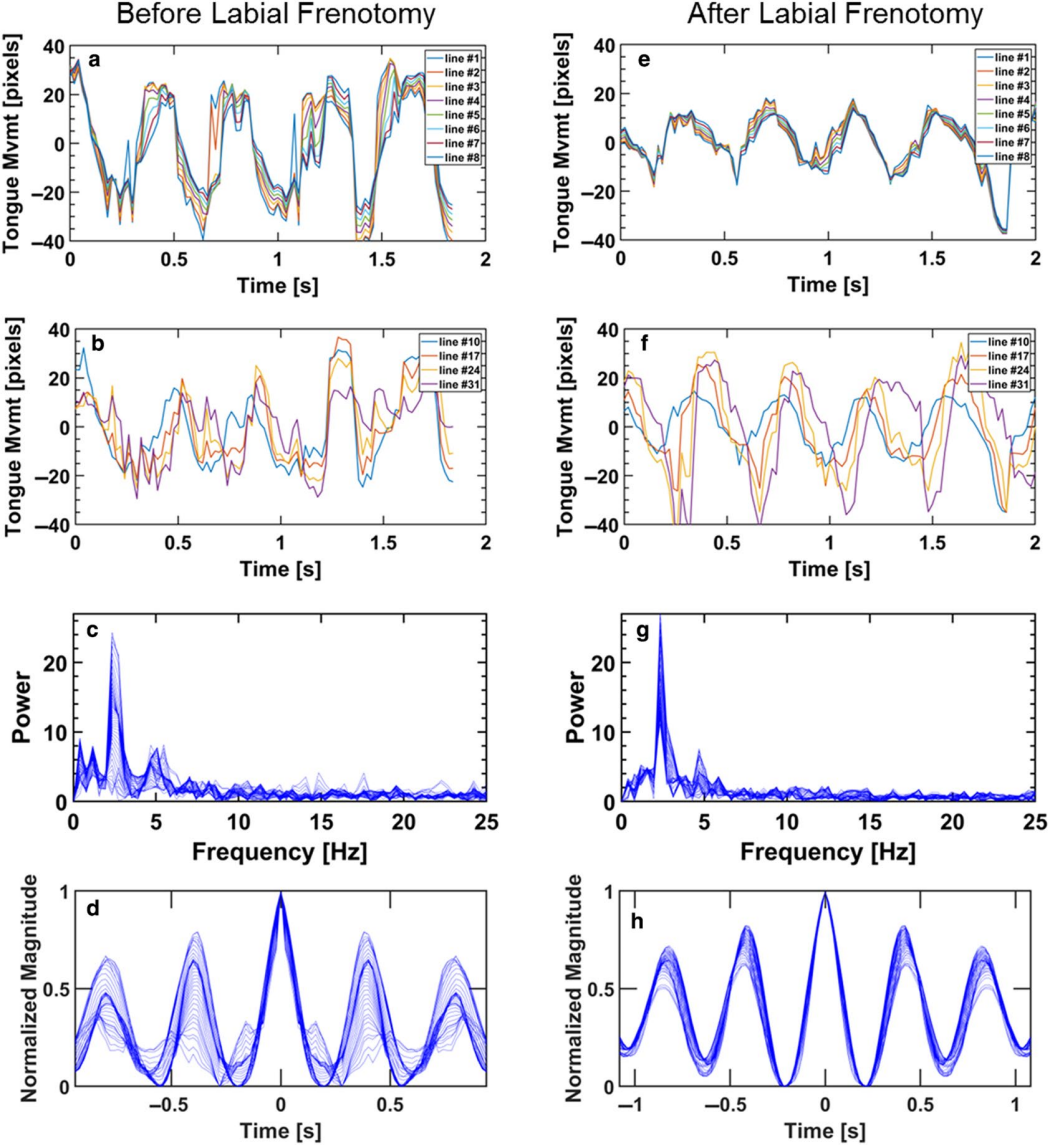
Tongue motility of infant with a restrictive superior labial frenulum before (a through d) and after labial frenotomy (f through h). (a,e) Motility of the anterior tongue pre‐ and post‐frenotomy, (b,f) Motility of the posterior tongue pre‐ and post‐frenotomy, (c,g) Frequency distribution of tongue motility pre‐ and post‐frenotomy, (d,h) Periodicity (autocovariance) of tongue motility pre‐ and post‐frenotomy

The kinematics of tongue motility of a 6.5‐week‐old infant with a restrictive superior labial frenulum before and after the upper lip frenotomy is shown in Figure 5. The movies with the tracked outlines of the tongue and palate are included in the supplemental Video S3. Before the surgical intervention, the movements of the anterior and posterior sections of the tongue have a noisy periodicity of a relatively dominant frequency, but without a peristaltic pattern for the posterior part (Figure 5a‐d). The pattern after labial frenotomy demonstrates definite improvement in tongue periodicity with a dominant frequency and peristaltic pattern for the posterior part (Figure 5e‐h). The pattern after frenotomy resembles that of a healthy infant. The Morlet wavelet analysis for both the tongue‐tied and lip‐tied infants, before and after frenotomy, also revealed the improvement of the time‐frequency spectrum of both the anterior and posterior tongue (See supplemental Figs. S1 and S2.

**Figure 6:**
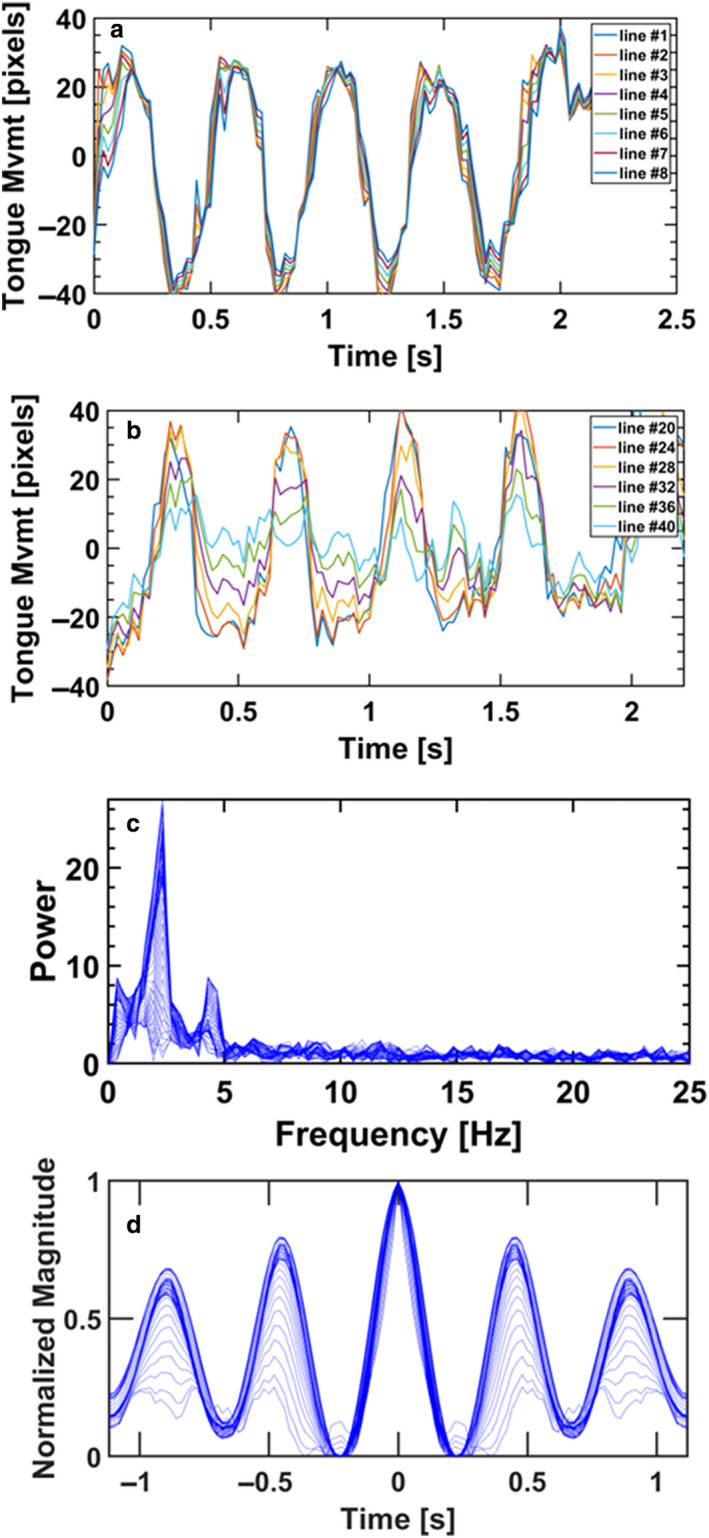
Tongue motility of infant with dysphagia during breast feeding. (a) Motility of the anterior tongue, (b) Motility of the posterior tongue, (c) Frequency distribution of tongue motility, (d) Periodicity (autocovariance) of tongue motility

We also recorded and analyzed the tongue motility during the breastfeeding of a 12‐week‐old infant with dysphagia secondary to placental abruption. The results are summarized in Figure 6 and demonstrate beautiful periodicity with a dominant frequency of 2.34 Hz. However, the scaled motility of both anterior and posterior parts of the tongue (Figure 6a,b) demonstrates rigid body motility. While this type of pattern is functional for the anterior part to stimulate the nipple‐areola complex, it is insufficient for the posterior part which indicates the impaired function of the mechanism required for swallowing the milk extracted from the breast. The movies with the tracked outlines of the tongue and palate can be seen in supplemental Video S4.

**Figure 7:**
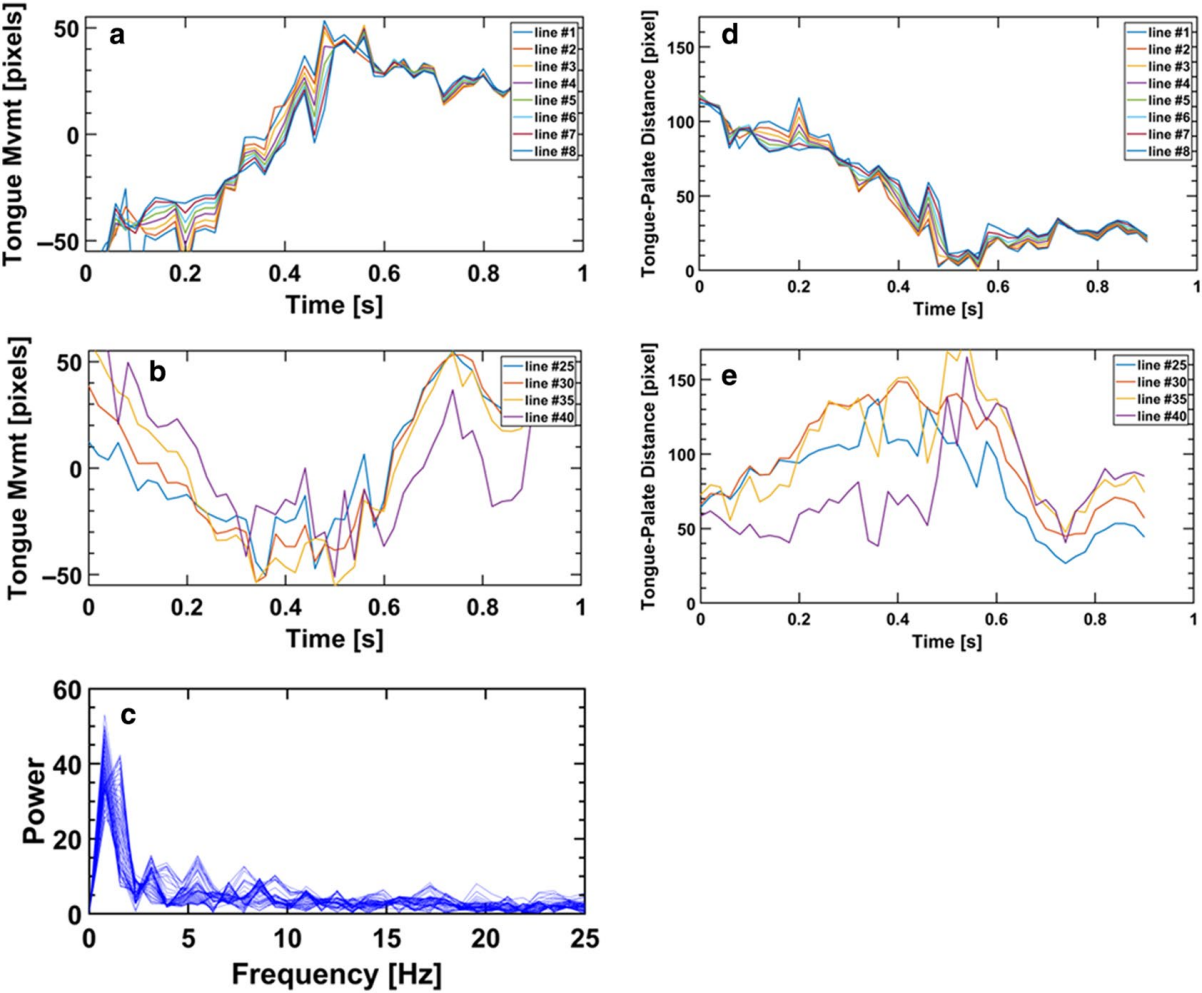
Tongue motility of an adult during swallowing a bolus of water. (a) Motility of the anterior tongue, (b) Motility of the posterior tongue, (c) Frequency distribution of tongue motility, (d) Gum‐Tongue distance of the anterior tongue, (e) Gum‐Tongue distance of the posterior tongue

Finally, we applied the objective analysis of tongue motility to ultrasound video clips acquired in a healthy adult while swallowing a bolus of water. The anterior tongue motility of anterior and posterior segments of the tongue is depicted in Figure 7 along with the frequency spectrum about each of the polar coordinates. Inspection of the video clip after the analysis (see the supplemental Video S5 clearly demonstrates how the anterior tongue is moving toward the palate to close the oral cavity which is complemented by the upward moving of the posterior part to drive the bolus into the oropharynx. This quick maneuver is depicted by the peristaltic motion of the anterior part (Figure 7a) which is followed by the peristaltic motion of the posterior part (Figure 7b). The instantaneous distance between the tongue and palate is depicted in Figure 7 d and e for the anterior and posterior tongue, respectively.

## 4 DISCUSSION

The tongue is a unique, single‐muscle organ of interdigitated muscle fibers (Takemoto, 2001) which plays essential roles in feeding and speech. The literature of the past decades presents an overwhelming need for novel protocols to evaluate irregularities in tongue function in both children and adults (Bahia & Lowell, 2020; Delaney & Arvedson, 2008). In the present work, we demonstrated how the objective and quantitative analysis of tongue motility (Elad et al., 2014) can be used to highlight differences in tongue motility during disease states, different methods of feeding, and before and after surgical interventions to improve tongue performance.

The first example of the infant with left torticollis and airway instability is the first demonstration comparing the physiology of the tongue in the same infant in both breast‐ and bottle‐feeding (Figure 3). While this infant has left torticollis and airway instability, the observed motility during breastfeeding is similar to the motility seen in healthy infants (Elad et al., 2014), but the lines are more smeared, representing poorer rhythmicity. Congenital muscular torticollis has been clinically associated with weaker sucking (Genna, 2015; Kaplan et al., 2018), and though this infant transfers milk from the breast, they require 25% of feedings as expressed milk via bottle to grow well. The anterior tongue moves as a rigid body against the nipple‐areola complex to induce the milk ejection reflex and to stabilize the breast in the mouth, while the posterior part undulates in a peristaltic pattern downward to reduce intraoral pressure to extract milk, and upward to swallow the extracted maternal milk. The measured motility during bottle‐feeding is the first instance where different patterns of tongue motility for breast‐ and bottle‐feeding can be distinguished. While the anterior and posterior sections of the tongue exhibit smoother and repeatable periodic movements during breastfeeding, during bottle‐feeding, the motion is variable without a distinct periodicity. This major difference is observed in the frequency distributions (Figure 3c and h), the periodicity (Figure 3 d and i), and the Morlet wavelet analysis comparing local motility in adjacent polar lines (Figure 3 e and j).

The literature is rich with observations and studies of the differences between breast‐ and bottle‐feeding. These studies highlight the disadvantages of bottle‐feeding, especially the problem of nipple confusion: changes in feeding behavior that make breastfeeding more difficult after exposure to bottles (Batista et al., 2019; Mizuno & Ueda, 2006; Moral et al., 2010; Praborini et al., 2016). However, the observations are based on subjective descriptions of the tongue and orofacial muscles, specifically using visual analysis of videofluoroscopy swallow (Hernandez & Bianchini, 2019), EMG measurement of facial muscles (França et al., 2014), recording of swallowing sounds (Tamura et al., 1996), and rates of sucking and breathing (Taki et al., 2010). The present analysis of ultrasound video clips elucidates the dynamic pattern of the infant's tongue during feeding. The results of Figure 3 demonstrate that the overall frequency spectrum and pattern of the undulation of the tongue during breastfeeding and bottle‐feeding are similar. However, the natural infant‐mother biomechanical compatibility that exists in breastfeeding is missing in bottle‐feeding due to differences between the mechanical characteristics (e.g., size, stiffness, conformability, viscoelasticity) between the artificial nipple and the maternal nipple‐areola complex. During bottle‐feeding, the tongue attempts to perform its natural motility, as during breastfeeding, but the mismatch of characteristics forces the tongue to perform variable, chaotic kinematics.

The next examples highlight pathologies that disrupt the natural motion of the tongue during feeding, either directly or by limiting the amount of breast tissue that can be maintained in the mouth (i.e., latch), specifically tongue‐tie and lip‐tie, respectively (Figures 4 and 5). In both examples, the surgical interventions, either lingual or labial frenotomy, clearly affected tongue kinematics allowing the tongue to move with a pattern more similar to the healthy breastfeeding infant after surgery (Elad et al., 2014). The effects of surgery could be seen by the fact that the post‐frenotomy motility has a much clearer dominant frequency (Figures 4c,g and 5c,g) and the movement of the tongue post‐surgery had a smooth periodicity along most of the tongue (Figures 4d,h and 5d,h). Currently, the severity of such pathologies (i.e., tongue‐tie and lip‐tie) are determined via assessments derived from observational criteria (Baeza et al., 2017; Ghaheri et al., 2017; Martinelli et al., 2012) and there is great controversy over when frenotomy is necessary (O'Shea et al., 2017; Power & Murphy, 2015). Therefore, an objective analysis of tongue‐motility, such as the method applied here, may be a useful addition to the diagnostic and follow‐up toolbox.

Upper lip frenotomy is poorly studied (Ghaheri et al., 2017; Nakhash et al., 2019). There are only small samples reporting variable rates of the maternal perception of improvement after isolated superior labial frenotomy (Benoiton et al., 2016). It is likely that the improved tongue motility seen after the treatment of the restrictive superior labial frenulum in our sample was attributable to improved latch, as infants with a tight upper lip tend to repeatedly slip down to the nipple. Cine‐MRI imaging of 11 breastfeeding infants demonstrated that the upper lip was most commonly neutral and slightly flanged in only two infants (Mills et al., 2020). This suggests that the upper lip mobility necessary for breastfeeding may be overestimated.

Analysis of tongue motility of an infant with dysphagia during breastfeeding (Figure 6) provides additional support for the value of dynamic analysis of tongue motility. Dysphagia is a complex disability that may involve four compartments of the digestion system: the oral cavity, pharynx, esophagus or the gastroesophageal junction. Here, we demonstrated a methodology to more objectively evaluate the role that the tongue plays in such patients. The results revealed smooth motility with a dominant frequency and good periodicity. However, the peristaltic undulation is absent in the posterior part of the tongue which is normally responsible for forming a bolus moving it to the pharynx. Most esophageal disorders require specific, individual tests for diagnosis; for example, biopsy is required to distinguish eosinophilic esophagitis from esophageal dysphagia. The present methodology can quantitatively and objectively identify oropharyngeal dysphagia and may also be used to evaluate treatment interventions.

In this work, we also imaged a healthy adult swallowing a 5 ml bolus of water. This rapid maneuver of about 0.5 second is illustrated for the first time in Figure 7. Our objective time‐dependent measurements show the anterior tongue moving to the palate to seal the oral cavity, enclose the bolus and move it toward the depressed posterior tongue, which then elevates in a peristaltic‐like movement to propel the bolus to the pharynx. Unlike breastfeeding or bottle‐feeding where the anterior tongue is interposed between the mandibular gum ridge and the teat and moves as a unit, the anterior tongue in adult swallowing moves in a peristaltic pattern (Figure 7). This suggests that the rigid movement pattern of the anterior tongue during breastfeeding reflects the need to stabilize the breast in the mouth, while sealing of the anterior oral cavity is achieved by the infant's latch. It should be noted that both in infants and adults the posterior tongue moves upward in a peristaltic‐like motion to execute the oral phase of swallowing during feeding.

The tongue is the major organ in the initiation of digestion in the oral cavity. A functional tongue maintains the food within the oral cavity, moves it to the chewing surfaces of the teeth, forms it into a bolus and finally, propels it posteriorly into the oropharynx. The timing of the last stage of posterior propulsion of the food is coordinated with laryngeal closure to avoid food penetration into the pulmonary airways. Many studies measured the tongue‐palate contact force and demonstrated decreased tongue strength with age and associated with dysphagia (Peladeau‐Pigeon & Steele, 2017). While advancing the knowledge of motor control and functional stability, the impact of these changes in tongue undulation and swallowing physiology is not well understood (Steele & Huckabee, 2007). Tongue strength has been shown to improve with tongue resistance training exercises (Kim et al., 2017). However, inconsistent improvements on swallowing parameters across studies in a systematic review call for future efforts to analyze swallowing kinematics (Smaoui et al., 2019).

It is likely that the reduced tongue strength associated with dysphagia in stroke victims stems from central neurological mechanisms that also impede normal motility, rather than being the sole cause of poor swallowing. Children with dysphagia secondary to muscle atrophy in muscular dystrophy perform better on thin liquids than thicker foods that take more muscle action to propel to and through the pharynx (van den Engel‐Hoek et al., 2017), which is opposite to patients with neurologically based dysphagia, even though both have reduced tongue strength. Children with neuromuscular disorders have an increased risk of pharyngeal residue, whereas those with cerebral palsy show difficulties with every phase of swallowing (van den Engel‐Hoek et al., 2014). Motor learning, re‐routing around damaged brain areas, or similar beneficial effects on the central nervous system can co‐occur, particularly if functional exercises are included, such as saliva swallowing (Steele et al., 2016). The methodology presented here provides an objective way to study tongue kinematics during oral feeding and assess tongue motility improvements in future studies on dysphagia rehabilitation. Recent attempts to analyze tongue motility using cine‐MRI during breastfeeding were impeded by low resolution (Mills et al., 2020).

We saw the most organized and rhythmic tongue kinematics in infants who were exclusively breastfeeding, poorer motility in those with conditions that impede normal feeding such as tongue‐tie and more disorganized motility in bottle‐feeding than breastfeeding. This highlights the pivotal role of tongue kinematics in suckling from the breast or sucking from a bottle. The infant with neurologically based dysphagia was able to use the tongue rhythmically but without the undulation required for peristaltic‐like motility. He was able to extract milk, but was fed by a gastrostomy tube due to his history of aspiration, and allowed brief breastfeeding to maintain interest and ability in oral feeding. This case illustrates the importance of posterior tongue peristaltic‐like motility in the coordination of suckling, swallowing, and breathing. Many publications focus on the normal and abnormal development of infant and pediatric feeding and swallowing skills (Delaney & Arvedson, 2008; van den Engel‐Hoek et al., 2017; Sasegbon & Hamdy, 2017). While the descriptions are commonly abstractive, efforts were made to explore muscle strength and electrical activity, including the response to rehabilitation exercises. Ability to track the dynamic motility of the tongue during feeding can add to the objective tools available to track progress in pediatric rehabilitation.

A deep attachment to the breast is vital to the stability of the infant's oral structures and subsequent milk transfer (Mizuno et al., 2008); thus, each dyad was assisted to achieve the best latch possible before each scan. The presence of the ultrasound probe may reduce postural stability by coming between the mother and infant; or conversely assist the infant by supporting the sublingual muscles. Care was taken to use similar positions and pressure before and after treatment to at least ensure these variables were comparable. Other limitations may include the need for high framerate, high‐resolution ultrasound images captured without disrupting the infant's feeding. A far‐field thin film probe with a curvilinear array would allow data acquisition without interposing the long stem of the intracavity probe between the mother and infant. This would also facilitate the study of a wider variety of positioning interventions for safer swallowing. Attachment to the breast is a major confounder, as it determines tongue stability and affects motility. Synchronous videotaping of the infant at breast during ultrasound acquisition is recommended as a next step to ascertain the relative contributions of attachment and anatomical factors on tongue motility in infants with various conditions impacting feeding.

## 5 CONCLUSIONS

At present, it is a challenge to distinguish among the many pathologies of swallowing due to the absence of quantitative tools. We objectively quantified tongue kinematics from ultrasound movies non‐invasively acquired during eight conditions of infant and adult feeding. Differences in tongue motility were observed before and after treatment in infants with tongue‐tie and restrictive labial frenum. An infant with torticollis was imaged for the first time both during breast‐ and bottle‐feeding, showing slightly less rhythmic sucking than typical during breastfeeding, but arrhythmic sucking during bottle‐feeding. An infant with dysphagia was able to extract milk from the breast, but displayed an absence of posterior tongue peristalsis required for safe swallowing. Full tongue peristaltic‐like movements were identified during water bolus swallowing in a healthy adult, with simultaneous anterior oral cavity closure and posterior tongue depression to shape and hold the bolus before the posterior tongue propelled the bolus to the pharynx. Infant breastfeeding follows the same kinematic pattern of tongue movement except for the anterior tongue which supports the breast begins the movement cascade with stiff movement, followed by the peristaltic movement of the posterior tongue. The present methodology of quantitative imaging, even though it was demonstrated on a small group of subjects, revealed highly conserved patterns of tongue kinematics in infants and adults that can be used to differentiate between swallowing pathologies may also be used to evaluate treatment interventions. This method is novel and objective, and has the potential to advance knowledge about normal feeding and management of feeding disorders.

## CONFLICT OF INTEREST

No conflicts of interest, financial or otherwise, are declared by the authors.

## AUTHOR CONTRIBUTIONS

D.E., C.W.G., and A.F.L. conceived and designed the research; C.W.G, SAS, and Y.S. performed experiments; Y.S., C.W.G, and D.E. analyzed the data; D.E., C.W.G, Y.S., and A.F.L. interpreted results of experiments; Y.S and D.E. prepared figures; D.E., C.W.G, and Y.S. drafted the manuscript; D.E., C.W.G, Y.S., S.A.S, and A.F.L. edited and revised the manuscript; D.E., C.W.G, Y.S., S.A.S, and A.F.L. approved the final version of the manuscript.

## Supporting information



Supplementary Material
