# Supplementary material for: Quantitative imaging of tongue kinematics during infant feeding and adult swallowing reveals highly conserved patterns
Source: Physiol Rep. 2021 Feb 6;9(3):e14685. doi: 10.14814/phy2.14685 (PMC7866619; doi:10.14814/phy2.14685)
Supplement: Supplementary file 1 — Supplementary Material [file PHY2-9-e14685-s001.zip › RE__PHY2_14685__PHY2-2020-11-0480.R1_-_Supplemental_Material (1).zip!/Supplemental Figures-21-September-2020.docx]

**Quantitative Imaging of Tongue Kinematics during Infant Feeding and Adult Swallowing Reveals Highly Conserved Patterns**

Catherine W Genna, Yiela Saperstein, Scott A Siegel, Andrew F Laine, David Elad

**Supplemental Figures**

**Figure S1:** Tongue motility of tongue-tied infant before (a) and after lingual Frenotomy (b). Coherence of pairs of motility signals during breast- and bottle-feeding at in the anterior (lines 5 & 7), middle (lines 8 & 12) and posterior tongue (lines 17 & 21).

**Figure S2:** Tongue motility of infant with a restrictive superior labial frenulum before (a) and after lingual Frenotomy (b). Coherence of pairs of motility signals during breast- and bottle-feeding at in the anterior (lines 5 & 7), middle (lines 8 & 12) and posterior tongue (lines 17 & 21).
