# Supplementary material for: Quantitative imaging of tongue kinematics during infant feeding and adult swallowing reveals highly conserved patterns
Source: Physiol Rep. 2021 Feb 6;9(3):e14685. doi: 10.14814/phy2.14685 (PMC7866619; doi:10.14814/phy2.14685)
Supplement: Supplementary file 1 — Supplementary Material [file PHY2-9-e14685-s001.zip › RE__PHY2_14685__PHY2-2020-11-0480.R1_-_Supplemental_Material (1).zip!/Supplemental Video Legends-21-September-2020.docx]

**Quantitative Imaging of Tongue Kinematics during Infant Feeding and Adult Swallowing Reveals Highly Conserved Patterns**

Catherine W Genna, Yiela Saperstein, Scott A Siegel, Andrew F Laine, David Elad

**Supplemental Videos**

**Legends**

| Video S1. | Tongue motility of infant with torticollis during breast- and bottle-feeding. |
| --- | --- |
| Video S2. | Tongue motility of tongue-tied infant before and after lingual frenotomy. |
| Video S3 | Tongue motility of infant with a restrictive superior labial frenulum before and after lingual Frenotomy. |
| Video S4 | Tongue motility of infant with dysphagia during breastfeeding. |
| Video V5 | Tongue motility of an adult during swallowing a bolus of water. |
